# Chlorfenapyr metabolism by mosquito P450s associated with pyrethroid resistance identifies potential activation markers

**DOI:** 10.1038/s41598-023-41364-2

**Published:** 2023-08-29

**Authors:** Cristina Yunta, Jocelyn M. F. Ooi, Folasade Oladepo, Sofia Grafanaki, Spiros. A. Pergantis, Dimitra Tsakireli, Hanafy M. Ismail, Mark J. I. Paine

**Affiliations:** 1 Liverpool School of Tropical Medicine, Liverpool, L3 5QA UK; 2 Department of Chemistry, University of Crete, Voutes Campus, Heraklion, 700 13 Greece; 3 Institute of Molecular Biology and Biotechnology, Foundation for Research and Technology, Hellas, 100 N. Plastira Street, Heraklion, 700 13 Greece; 4 Laboratory of Pesticide Science, Department of Crop Science, Agricultural University of Athens, 75 Iera Odos Street, Athens, 118 55 Greece

**Keywords:** Biochemistry, Entomology

## Abstract

Chlorfenapyr is a pro-insecticide increasingly used in combination with pyrethroids such as a-cypermethrin or deltamethrin in insecticide treated bednets (ITNs) to control malaria transmitted by pyrethroid-resistant mosquito populations. Chlorfenapyr requires P450 activation to produce tralopyril and other bioactive metabolites. Pyrethroid resistance is often associated with elevated levels of chemoprotective P450s with broad substrate specificity, which could influence chlorfenapyr activity. Here, we have investigated chlorfenapyr metabolism by a panel of eight P450s commonly associated with pyrethroid resistance in *An. gambiae* and *Ae. aegypti,* the major vectors of malaria and arboviruses. Chlorfenapyr was activated to tralopyril by An. *gambiae* CYP6P3, CYP9J5, CYP9K1 and *Ae. aegypti*, CYP9J32. The K_cat_/K_M_ value of 0.66 μM^−1^ min^−1^ for CYP9K1 was, 6.7 fold higher than CYP6P3 and CYP9J32 (both 0.1 μM^−1^ min^−1^) and 22-fold higher than CYP9J5 (0.03 μM^−1^ min^−1^). Further investigation of the effect of -cypermethrin equivalent to the ratios used with chlorfenapyr in bed nets (~ 1:2 molar ratio) resulted in a reduction in chlorfenapyr metabolism by CYP6P3 and CYP6K1 of 76.8% and 56.8% respectively. This research provides valuable insights into the metabolism of chlorfenapyr by mosquito P450s and highlights the need for continued investigation into effective vector control strategies.

## Introduction

Synthetic pyrethroids are the most widely used insecticides for vector control due to their knockdown effect, excito-repellency properties and low mammalian toxicity^1^. However, the increasing use of pyrethroids in vector control operations has resulted in a widespread occurrence of species that are resistant to pyrethroid insecticides^2^. For a long time, the range of available insecticides for vector control was limited to only a few classes, organochlorines, organophosphates, carbamates and pyrethroids. However, to address the increased numbers of insecticide-resistant vectors and non-target organism risks, new active ingredients with unique modes of action are being developed to ensure the sustainability and effectiveness of vector control strategies^3,4^.

Chlorfenapyr, a pyrrole insecticide (IRAC group 13) that impairs mitochondrial activity and has been widely employed in agricultural and urban pest control since 1995^5^, is one of the first of a new generation of malaria preventative products being developed by BASF with support from the Innovative Vector Control Consortium (IVCC). Chlorfenapyr is a highly potent insecticide with low repellent efficacy but high residual toxicity to mosquitoes, making it an attractive choice for combination with a pyrethroid to improve net user protection and reduce resistance selection.

In 2017, long lasting insecticide treated nets (LLINs) developed by BASF (Interceptor^®^ G2 LLIN) with dual ingredients (α-cypermethrin and chlorfenapyr) were prequalified by the World Health Organization (WHO) as first in class for dual insecticidal nets. Interceptor^®^ G2 LLIN is specifically designed to efficiently combat resistant mosquitoes, thereby safeguarding public health^6^. Large-scale trials in Benin and Tanzania demonstrated that Interceptor^®^ G2 LLIN reduced child malaria incidence by 46% and 44%, respectively, over 2 years compared to standard pyrethroid-only nets^7,8^. This resulted in a recent WHO recommendation published in March 2023 for the deployment of pyrethroid-chlorfenapyr LLINs instead of pyrethroid-only nets to prevent malaria in adults and children in areas where mosquitoes have become resistant to pyrethroids. The increase in demand for these nets has sparked the development of other varieties of pyrethroid-chlorfenapyr nets, such as PermaNet^®^ Dual, which contains deltamethrin-chlorfenapyr, and has been recently added to the list of Prequalified Vector Control Products.

**Figure 1:**
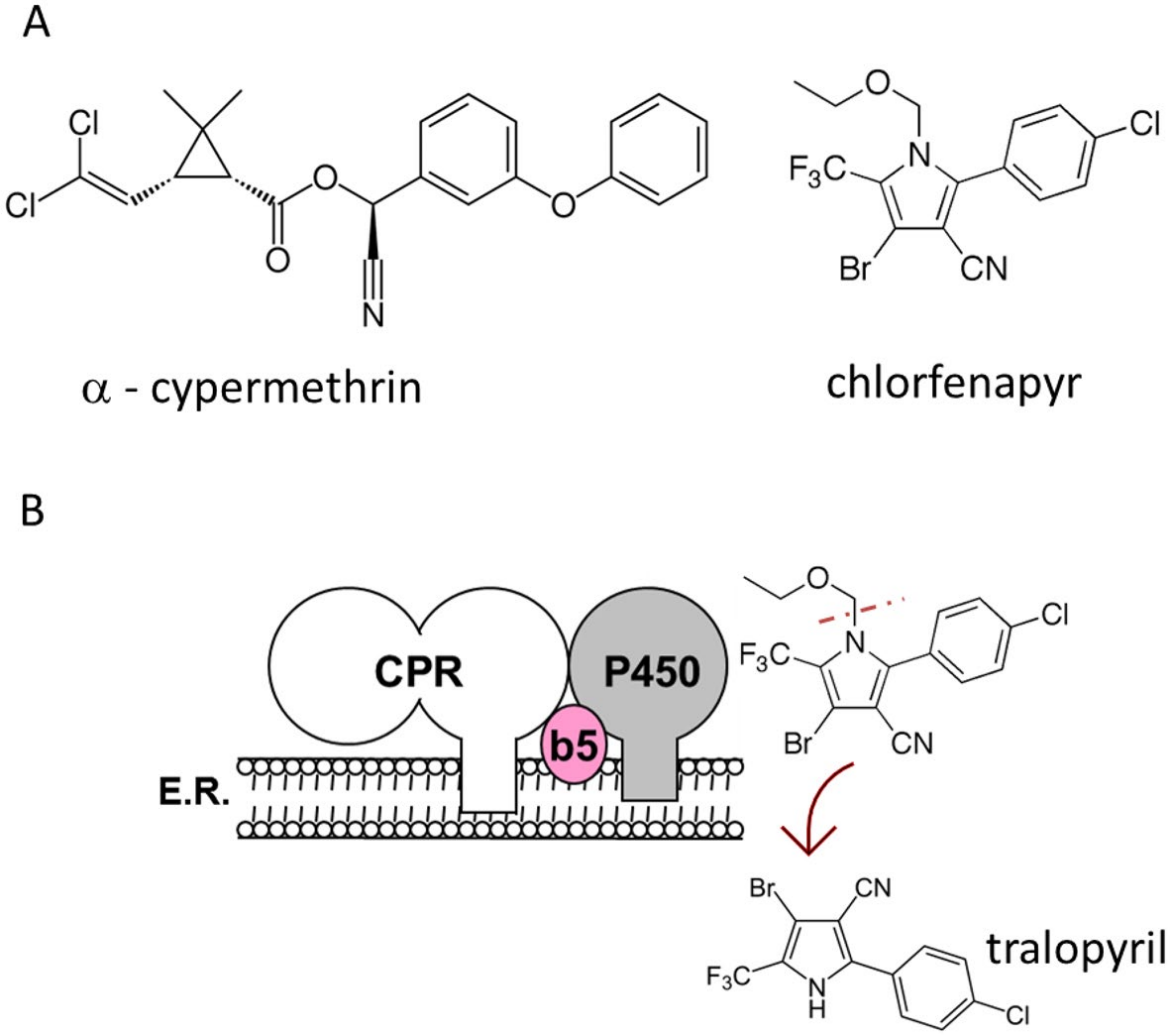
Scheme of chlorfenapyr N-dealkylation mediated by P450. (**A**) insecticides used in the study and (**B**), P450 oxidation produces tralopyril, a toxic N-dealkylated metabolite .

Chlorfenapyr is a pro-insecticide which becomes toxic when the N-ethoxymethyl group is removed through P450-mediated oxidation. This process creates the toxic metabolite tralopyril (also called the CL303268 metabolite). (Fig. 1). Tralopyril is a mitochondrial electron transport uncoupler (METU) whose mode of action is to disrupt the proton gradient across the mitochondrial membranes and impairs the production of ATP (oxidative phosphorylation)^5,9^ leading to cell death. The mode of action of chlorfenapyr differs significantly from that of standard neurotoxic insecticides^10,11^ raising expectations for minimal cross–resistance issues.

Constant selection pressure from pyrethroids used in bed nets and indoor residual spraying (IRS) has produced widespread pyrethroid resistant populations of malaria transmitting Anophelines across the African continent^12^. Consequently, constitutively elevated levels of P450s associated with pyrethroid metabolism and insecticide resistance are commonly present in African mosquito species. These include CYP6M2, CYP6P2, CYP6P3, CYP6P4, CYP6P5, CYP9K1 and CYP9J5^13–19^ from *An. gambiae*, CYP6P9a, CYP6P9b, and CYP6M7 from *An. funestus*^20,21^*.* Elevated levels of pyrethroid metabolizing P450s are also found in *Ae. aegypti*, the vector for dengue and zika viruses, which include CYP9J32, CYP9J24, CYP9J26 and CYP9J28^22,23^. Given that chlorfenapyr is being introduced to populations of mosquitoes with high levels of P450s primed for xenobiotic metabolism, it is important to determine to what extent, if any, they might play a role in chlorfenapyr activation or detoxification. In this paper,we have screened chlorfenapyr against eight P450s that metabolise pyrethroids and are commonly associated with pyrethroid resistance in *An. gambiae* (CYP6M2, CYP6P2, CYP6P3, CYP6P4, CYP6P5, CYP9K1 and CYP9J5) and *Ae. aegypti* (CYP9J32).

## Results

### Mosquito P450 metabolism profile of chlorfenapyr

**Figure 2:**
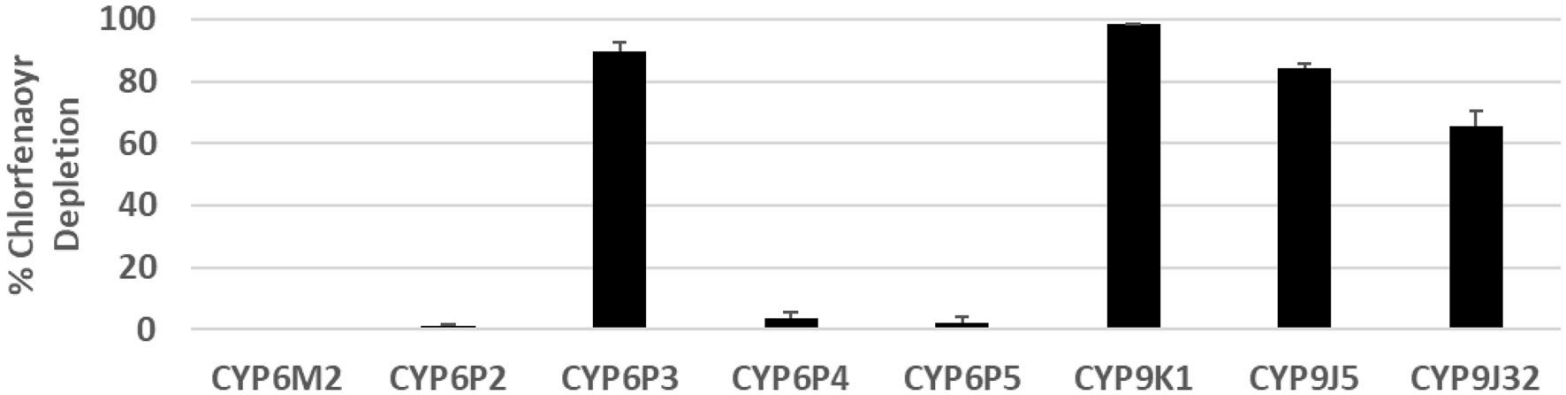
Chlorfenapyr metabolism by mosquito P450s. Bars represent the proportion (% depletion) of 10 μM insecticide cleared by 0.05 µM P450 in the presence of NADPH. Values are given in Supplementary Table 3. Error bars represent standard error (N = 3).

**Figure 3:**
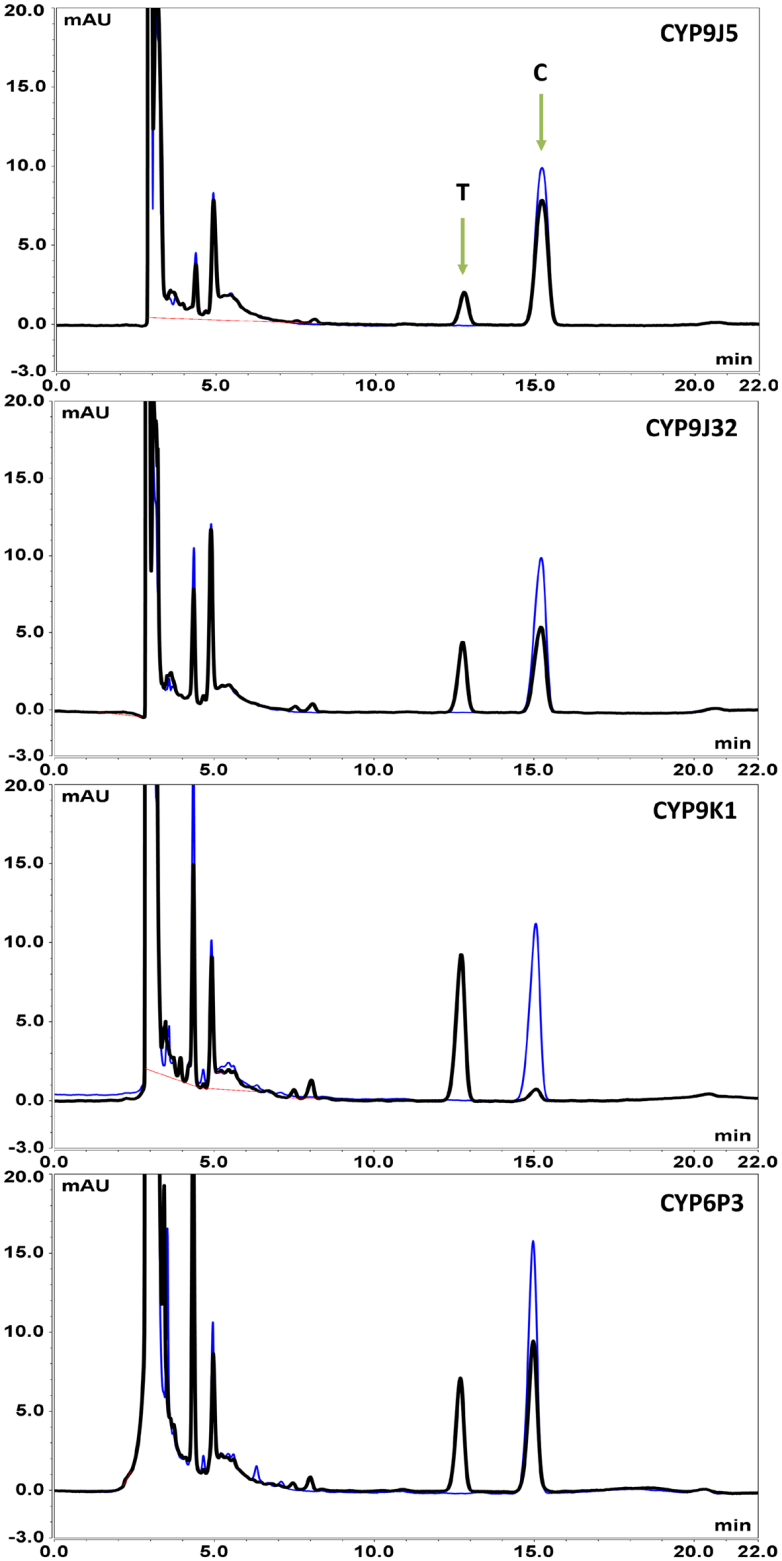
Representative HPLC chromatograms of chlorfenapyr metabolism by P450s. The overlaid chromatograms represent the results of 2 h incubations of 100 μl reactions containing a final concentration of 0.05 μM P450, 0.4 μM b5 and 10 μM compound in the presence (black) and absence (blue) of NADPH. Chlorfenapyr (C) and tralopyril (T) peaks are arrowed.

In order to examine the profile of chlorfenapyr metabolism, it was initially screened against seven *An. gambiae* P450s (CYPs 6M2, 6P2, 6P3, 6P4, 6P5, 9K1 and 9J5) and one *Ae. aegypti* P450 (CYP9J32). Four of the P450s were able to metabolize chlorfenapyr, *An. gambiae* CYP6P3, CYP9J5 and CYP9K1 and *Ae. aegypti* CYP9J32, as evidenced by chlorfenapyr depletion (Fig. 2 and Supplementary Table 1) and the production of a single NADPH dependent metabolite peak with a retention time corresponding to tralopyril (Fig. 3). The products of chlorfenapyr metabolism by CYP6P3, CYP9K1 and CYP9J32 were further investigated by LC–MS. Extracted ion chromatograms of [M + H]^+^ confirmed the presence of the insecticidal tralopyril (*m/z* 131.01) (Supplementary Fig. 1). Selected ion monitoring failed to identify any other chlorfenapyr metabolites produced by the P450s.

**Figure 4:**
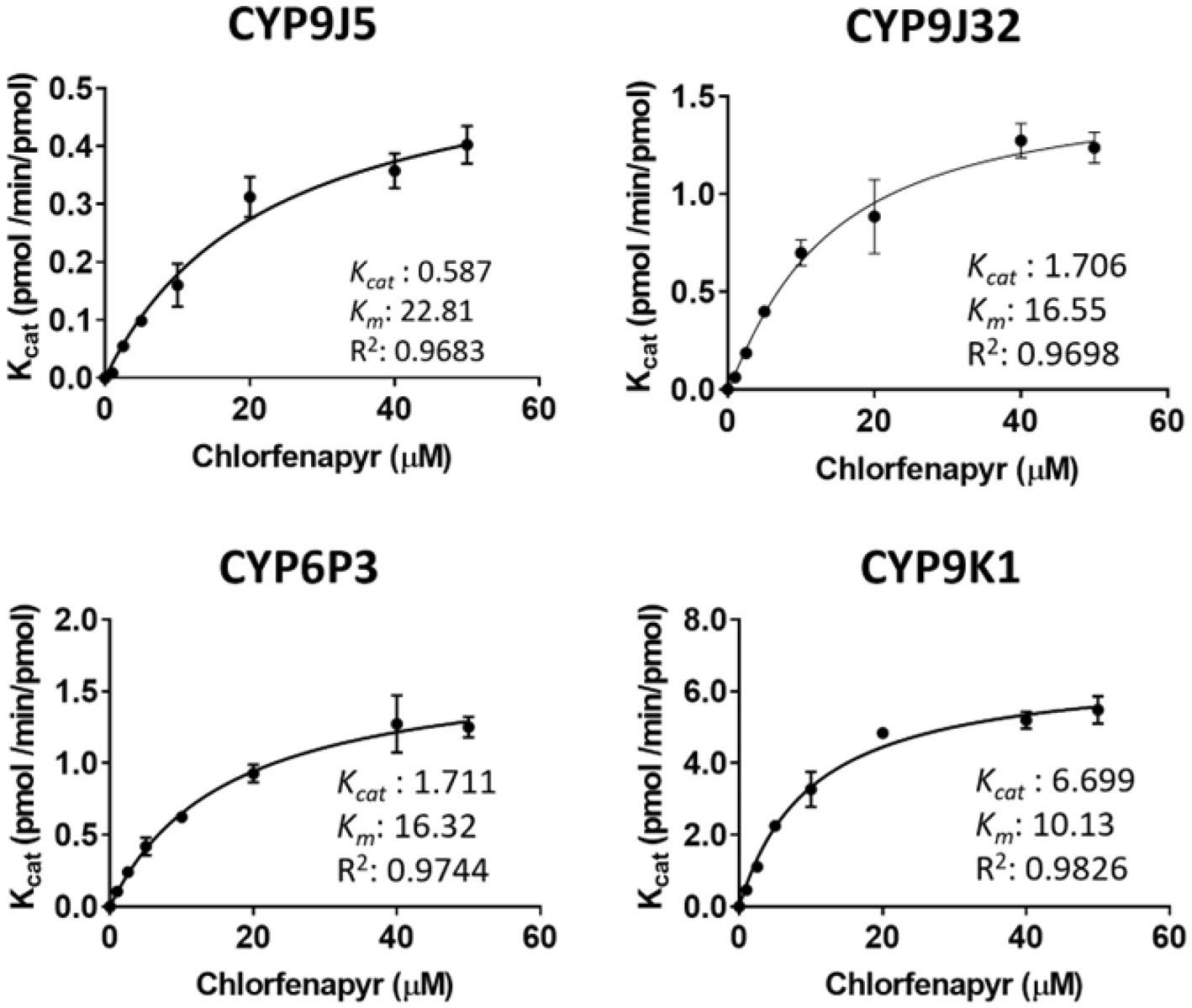
Steady-state enzyme kinetics of tralopyril formation by CYP9J5, CYP6P3, CYP9J32 and CYP9K1. Data are mean values +/− SD (n = 4). *K*_M_ and V_max_ values were calculated by nonlinear regression analysis using GraphPad Prism.

The steady-state kinetics of tralopyril production by CYP’s 6P3, 9J5, 9K1 and 9J32 were compared (Fig. 4 and Supplementary Table 2). Overall, the reactions followed Michaelis–Menten kinetics. CYP9K1 produced the highest rate of tralopyril production with a K_cat_ of 6.70 min^−1^, followed by CYP6P3 and CYP9J32 (both K_cat_ = 1.71 min^−1^) and CYP9J5 with a K_cat_ of 0.59 min^−1^. Similarly, CYP9K1 produced the lowest K_M_ value of 10.13 μM and CYP9J5 the highest K_M_ value of 22.81 μM. CYP’s 6P3 and 9J32 produced closely similar values of 16.32 and 16.55 μM respectively. Comparing the catalytic efficiencies of the enzymes (K_cat_/K_M_) (Supplementary Table 3), CYP9K1 was found to be the most efficient enzyme with a K_cat_/K_M_ value of 0.66 mM^−1^ min^−1^, 6.7 fold higher than CYP6P3 and CYP9J32 (both 0.1 μM^−1^ min^−1^) and 22-fold higher than CYP9J5 (0.03 mM^−1^ min^−1^).

### Interactions of chlorfenapyr and a-cypermethrin

**Figure 5:**
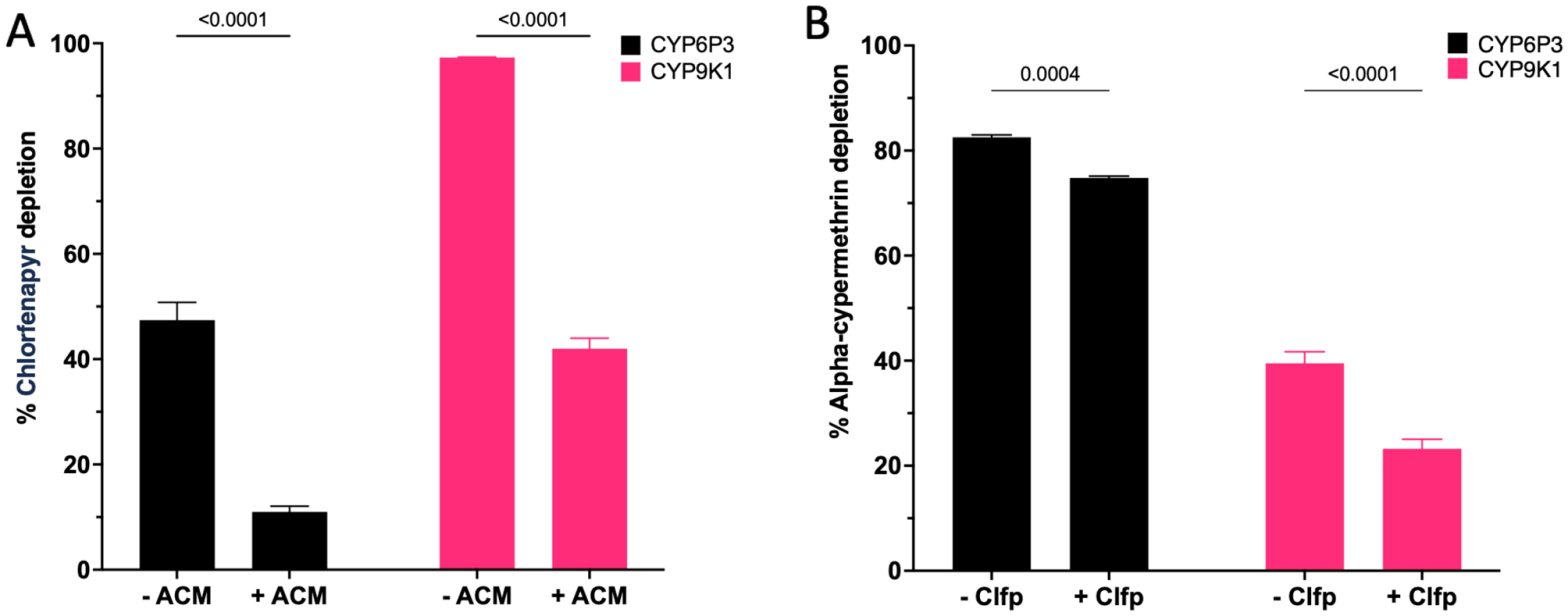
Mixture effects on chlorfenapyr and α-cypermethrin metabolism. Mixing chlorfenapyr with α-cypermethrin (ACM) in vitro has a negative effect on the activation metabolism of chlorfenapyr (Clfp) to its toxic metabolites, tralopyril. Panel (**A**) illustrates the percentage depletion of 20 µM Clfp cleared by 0.1 µM CYP6P3 and CYP9K1 (n = 3, *p* < 0.05) with 0.8 µM b5 in the presence of NADPH and ± 10 µM ACM, while panel (**B**) represents ACM (10 µM) clearance at the same conditions in the presence of 20 µM Clfp.

**Figure 6:**
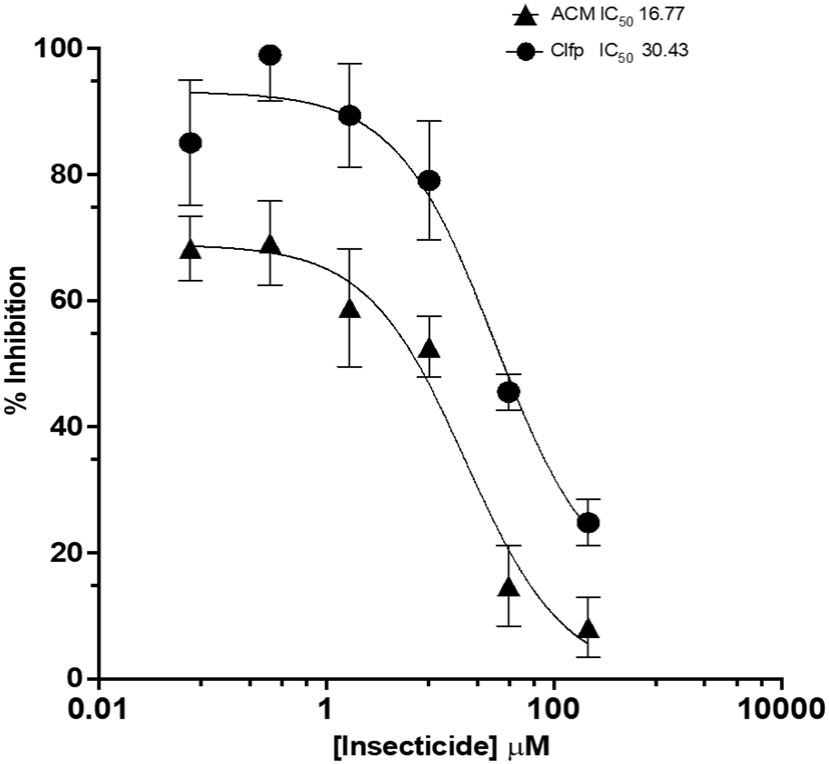
Determination of IC_50_ values for α-cypermethrin (ACM) and chlorfenapyr (Clfp) on CYP6P3 metabolism. Dose response analysis of ACM (closed triangles) and Clfp (closed circles) on P450 fluorescence substrate probe (DEF) metabolism.

Since chlorfenapyr (200 mg/m^2^) is being used in combination with a-cypermethrin (100 mg/m^2^) in ITNs, we investigated the effect of α-cypermethrin on the production of the toxic metabolite tralopyril by CYP6P3 and CYP9K1, the two *An. gambiae* P450s with highest activity. Chlorfenapyr metabolism by CYP6P3 and CYP6K1 was significantly inhibited by 76.9% and 56.8% respectively in the presence of α-cypermethrin at an equivalent ~ 1:2 molar ratio (Fig. 5A). By contrast, α-cypermethrin metabolism by CYP6P3 was minimally inhibited (9.3%) by chlorfenapyr, while CYP9K1 was similarly inhibited (41.2%) (Fig. 5B). The strong drop in CYP6P3 activity in the presence of α-cypermethrin coupled with the fact that this P450 is frequently elevated in pyrethroid resistant populations of *An. gambiae*^24^ led to further inhibition tests using the fluorescent probe substrate, DEF, where α-cypermethrin produced ~ two-fold stronger inhibition of DEF activity than chlorfenapyr (*IC*_50_ 16.7 vs 30.4 μM), indicating a stronger affinity for the active-site of CYP6P3 (Fig. 6).

## Discussion

Here, we have shown that the pro-insecticide chlorfenapyr is metabolized by three P450s that are commonly overexpressed in pyrethroid resistant populations of *An. gambiae* CYP6P3, CYP9J5, CYP9K1 and one in *Ae. aegypti*, CYP9J32^13,15,16,22,25^. HPLC and LC–MS/MS analysis indicates that the principal metabolite produced by these P450s was tralopyril, the N-dealkylated insecticidal form that disrupts oxidative phosphorylation^5,9^. This suggests that populations of pyrethroid resistant mosquitoes where these P450s are overexpressed may have an enhanced capacity to activate chlorfenapyr, potentially resulting in improved susceptibility to the pro-insecticide.

This is supported by several early field and laboratory studies in Benin, Tanzania, India and South Africa that have demonstrated that chlorfenapyr is effective at controlling pyrethroid resistant mosquito species including *An. gambiae*, *Cx. quinquefasciatus*, *An. arabiensis*, *An culicifacies*, *An. funestus, Ae. aegypti*, and *An. quadrimaculatus*^10,26–31^. Most recently, targeted indoor residual spraying of experimental houses with chlorfenapyr has proven effective against locally pyrethroid resistant *Ae. aegypti* (Merida, Mexico)^32^, while dual action bed nets containing pyrethroids α-cypermethrin or deltamethrin and chlorfenapyr have proven more effective against pyrethroid resistant *An. gambiae* in Burkina-Faso and Coted’Ivoire respectively^7,33^, where elevated levels of CYP6P3 are reported^34,35^.

Early studies have also indicated that chlorfenapyr appears to be more toxic to pyrethroid resistant pests such as the cattle horn fly, or the tobacco budworm,where pyrethroid resistance is based on elevated P450 activity^36,37^. However, caution must be applied in correlating metabolic pyrethroid resistance with chlorfenapyr activation given that only four of the eight P450s tested were capable of metabolizing chlorfenapyr and rates of metabolism differed widely. *An. gambiae* CYP9K1 was most effective in producing tralopyril (K_cat_, 6.70 min^−1^), while rates of production were ~ fourfold lower with CYP6P3 (K_cat_, 1.71 min^−1^) and *Ae. aegypti* CYP9J32 (Kcat_,_ 1.71) and 11 fold lower with CYP9J5 (K_cat_, 0.59 min^−1^).

While the P450s identified by our screening results may be considered activating markers of chlorfenapyr activity, the multiplicity of P450’s present in mosquito genomes (> 100) means it is feasible that chlorfenapyr may be susceptible to detoxification by other P450s, which if identified, should be considered potential metabolic resistance markers. To date reduced susceptibility in some pyrethroid resistant populations in the DRC, Ghana, and Cameroon has been reported^38^, although some caution is required as bioassay testing guidelines have since been updated by WHO to take account of the strong influence of testing conditions that can lead to interlaboratory variability^39^. Further work is needed to identify the full range of P450s that interact with chlorfenapyr and the active site determinants of chlorfenapyr activation and detoxification.

Widespread resistance to pyrethroids has driven the development of new dual action pyrethroid ITNs that incorporate insecticides with different modes of action, such as chlorfenapyr, or PBO, a synergist that is a broad-spectrum inhibitor of P450s that can negate metabolic detoxification to enhance pyrethroid activity. However, the inhibition of P450 activity could also inhibit the toxicity of pro-insecticides such as chlorfenapyr. Previous studies have shown extensive cross-reaction of Anopheline pyrethroid metabolizing P450s with the pro-insecticide pirimiphos-methyl, with potential to both inactivate or activate insecticidal activity^40^. Pirimiphos-methyl, an organophospate insecticide, is extensively used by malaria control programmes in Africa for indoor residual spray operations. Recent evidence from experimental hut trails in Benin indicates that PBO containing ITNs can reduce the efficacy of indoor residual spraying with pirimiphos‑methyl against pyrethroid‑resistant malaria vectors^41^, supporting recommendations by the WHO to avoid the deployment of pyrethroid-PBO ITNs in areas that have already been programmed for IRS with pirimiphos-methyl IRS. Given that all four mosquito P450s that metabolized chlorfenapyr produced the toxic metabolite, tralopyril, and previous work has shown PBO to antagonize chlorfenapyr activity in mosquitoes^31,42^, similar recommendations are applicable to chlorfenapyr and further studies are required to determine the optimal co-deployment of products containing PBO and chlorfenapyr.

Likewise, competitive interactions will affect the ability of cross-reactive P450s to detoxify or activate pyrethroids and chlorfenapyr respectively. Alpha-cypermethrin is the pyrethroid most commonly used in combination with chlorfenapyr in bed nets. Our data (Figs. 5 and 6) demonstrates that compound ratio mixtures equivalent to bed net dosing inhibits the metabolism of both compounds by *An. gambiae* CYP6K1 and CYP6P3. The mixture effects on chlorfenapyr metabolism were somewhat greater, consistent with a higher affinity for α-cypermethrin as measured by the lower IC_50_ value for CYP6P3 (Fig. 6). While this suggests that chlorfenapyr activity could be compromised by the presence of α-cypermethrin, this may be counterbalanced by the increased susceptibility of mosquitoes to α-cypermethrin through dampened P450 activity. Furthermore, mosquitoes are likely to contain other compensatory P450s capable of chlorfenapyr activation, potentially non-cross reactive with pyrethroids; thus complex pharmacokinetic factors must be considered that will influence the insecticidal outcome.

## Conclusions

A recent review of the potential of pro-insecticides for resistance management^43^ reveals chlorfenapyr to have a high capacity for negative cross-reactivity, being primarily effective against pyrethroid resistant insects. We have identified four pyrethroid metabolizing P450 enzymes that are often overexpressed in pyrethroid-resistant mosquito populations that can metabolise the pro-insecticide chlorfenapyr to generate tralopyril, a highly toxic molecule that interferes with oxidative phosphorylation. These data suggest that chlorfenapyr to be a viable option for managing pyrethroid-resistant mosquito populations. However, because not all P450s can metabolize chlorfenapyr and metabolic rates vary, the data must be interpreted with care. Furthermore, the general suppression of P450 activity by synergists such as PBO or more targeted inhibition of chlorfenapyr activating P450s including CYP6P3, CYP9K1, CYP9J5, and CYP9J32 by α-cypermethrin and other competitive substrates^40^ can influence the toxicity of chlorfenapyr. As a result, further studies are required to determine the full range of P450s that interact with chlorfenapyr and the active site determinants for chlorfenapyr activation and detoxification.

## Material and methods.

### P450 expression and bactosome preparation

P450s were expressed using pCWori + expression vector constructs as described previously for *An. gambiae* CYPs 6M2, 6P2, 6P3, 6P4, 6P5, 9J5^44^ and CYP9K1^45^ and *Ae. aegypti* CYP9J32^22^. *E. coli* membranes co-expressing P450 and *An. gambiae* NADPH cytochrome P450 oxidoreductase (AgCPR) were supplied by Cypex Ltd, UK (www.cypex.co.uk). Reactions were supplemented with *An. gambiae* cytochrome b5 (b5) supplied by Cypex Ltd, UK and prepared as described previously^19^.

The membrane samples were analyzed for P450 quality and content by 30-fold dilution in Spectrum Buffer and CO-difference spectroscopy^46^. Cytochrome c reductase activity was used to measure CPR content^47^ and protein content was estimated by Bradford assay. Samples were stored in aliquots at − 80 °C.

### Insecticide metabolism

To test for chlorfenapyr metabolism, P450s were incubated at 30 °C for 2 h in 200 µl reactions containing 10 µM chlorfenapyr, 0.1 µM P450, 0.8 µM cyt b5 in 200 mM Tris–HCl pH 7.4, and NADPH regeneration components (1 mM glucose-6-phosphate (G6P), 0.25 mM MgCl_2_, 0.1 mM NADP^+^ (absent -NADP+), and 1 U/mL glucose-6-phosphate dehydrogenase (G6PDH)). Reactions were carried out in triplicate with 1200 rpm orbital shaking and quenched by adding 200 µl methanol. Samples were then incubated with shaking as before for an additional 5 min before centrifuging at 13000 rpm for 5 min. 150 µl of the supernatant was then transferred to HPLC vials, stored at room temperature, and analyzed within 24 h. Chlorfenapyr was prepared as a working stock in ethanol and stored at − 20 °C; solvent content was 2% of the final reaction (v/v). Results were calculated as percentage depletion of the insecticide peak area in the presence of NADPH (+NADPH) versus absence of NADPH (−NADPH) to give a quantitative assessment of metabolism.

For kinetic measurements, catalytic activity was assessed by measuring tralopyril production using 200 µl reactions containing varying concentrations of chlorfenapyr (1 µM to 50 µM), 0.1 µM P450, 0.8 µM cyt b5 in 50 mM potassium phosphate buffer (KPB) at pH 7.4, and NADPH regeneration components (1 mM glucose-6-phosphate (G6P), 0.25 mM MgCl_2_, 0.1 mM NADP^+^, and 1 U/mL Glucose-6-phosphate dehydrogenase (G6PDH)). Reactions were performed in duplicate with two independent biological replicates and compared against a negative control with no NADP^+^.

### HPLC analysis

A standard curve of tralopyril (0.04 μM to 40 μM) was prepared to determine the assay detection limit. Samples were analyzed by reverse-phase high-pressure liquid chromatography, RP-HPLC (Ultimate 3000 series, Dionex). 100 µl of reaction supernatant was analyzed with a monitoring absorbance at 226 nm using a 5 µm, C18 column (250 × 4.6 mm) (Hypersil Gold, Thermo Scientific) and a mobile phase consisting of 70% methanol and 30% water containing 0.1% phosphoric acid. The system was run at a flow rate of 1 ml/min at 40 °C. The production of tralopyril was quantified by peak integration (Chromeleon software, Dionex) and the concentration was calculated against the prepared standard curve.

### LC–MS/MS experiments

We incubated 0.25 μM of recombinant CYP (CYP9K1, CYP9J32, CYP6M2 or CYP6P3) and 2 μM b5 in 100 μl Tris–HCl buffer. Reactions contained 20 µM chlorfenapyr and NADPH regeneration components (1 mM glucose-6-phosphate (G6P), 0.25 mM MgCl_2_, 0.1 mM NADP^+^, and 1 U/mL Glucose-6-phosphate dehydrogenase (G6PDH)). Reactions were incubated for 0 and 2 h at 30 °C with 1250 rpm orbital shaking and quenched by adding 100 µl acetonitrile. Samples were then incubated with shaking as before for an additional 30 min before centrifuging at 14000* g* for 5 min. The supernatant was then transferred to LC–MS vials and analyzed within 24 h. Reactions were performed in duplicate (two independent biological replicates) and compared against a negative control with no NADPH regenerating system to calculate substrate depletion.

The analytes were separated by a UniverSilHS C18 column (250 mm × 4.5 mm i.d, 5 μm, Fortis Tech.). The mobile phase consisted of 85% methanol (A) and 15% of 40 mM Ammonium Acetate in water (B). Elution was performed with an isocratic mode. The flow rate was 0.8 ml min^−1^. The eluate from the HPLC column was split and then introduced into the MS detector at the flow rate of 0.24 ml min^−1^. The injection volume was 10 μl. Analysis of chlorfenapyr and tralopyril was conducted on a TSQ Quantum (Thermo Scientific, USA) equipped with an electrospray ionization (ESI) source.

The ESI–MS/MS detection was performed in negative ion mode and the monitoring conditions were optimized for target compounds. The conditions were described as follows: The Spray Voltage was set at 3.5 kV, the Capillary Temperature was held at 320° C and Sheath Gas pressure, and Aux Gas pressure were set at 30 and 15 Arb respectively. The selected reaction monitoring (SRM) mode was operated for each compound. The same SRM transitions were used for Chlorfenapyr and Tralopyril (Supplementary Fig. 1). Chlorfenapyr was detected in negative ion mode, and not in the protonated molecular ion form, due to the loss of the N-ethoxymethyl group when introduced in the ESI source. Quantitation was possible after HPLC separation. All the parameters for SRM transitions were optimized to obtain the highest sensitivity (Supplementary Table 3).

### Inhibition

To determine the IC_50_ values of CYP6P3 and CYP9K1, varying concentrations of α-cypermethrin and chlorfenapyr were used as ligands, and diethoxy fluorescein (DEF) substrate was utilized at approximately the K_m_ value. Dimethyl sulfoxide (DMSO) was added as a solvent to dissolve the insecticide and DEF substrate. The final concentration of DMSO in the 200 μl enzyme reaction mixture was 2%. Each reaction was carried out in triplicate and incubated for 30 min at 30 °C in 50 mM KPB at pH 7.4, using opaque white 96-well (flat-based) plates. A DEF concentration of 1.0 μM was mixed with 0.1 μM CYP6P3, and three replicates of positive and negative control reactions were also performed for the CYP6P3/DEF combination. The reaction mixture contained 1 mM glucose-6-phosphate (G6P), 0.1 mM NADP^+^, and 0.25 mM MgCl_2_. NADP^+^ and G6P were excluded in the minus NADPH controls. The plate was read on a FLUO star Omega plate reader (BMG LABTECH) using an excitation wavelength of 482 nm and an emission wavelength of 520 nm. The *IC*_*50*_ values were determined using GraphPad Prism 9 by fitting the data to a dose–response model, and plots with R^2^ values below 0.95 were excluded.

### Supplementary Information

Supplementary Information.

## Data Availability

Raw data files are available upon request from mark.paine@lstmed.ac.uk and hanafy.ismail@lstmed.ac.uk.
