# Author Correction: Chlorfenapyr metabolism by mosquito P450s associated with pyrethroid resistance identifies potential activation markers

**DOI:** 10.1038/s41598-023-47249-8

**Published:** 2023-11-16

**Authors:** Cristina Yunta, Jocelyn M. F. Ooi, Folasade Oladepo, Sofia Grafanaki, Spiros. A. Pergantis, Dimitra Tsakireli, Hanafy M. Ismail, Mark J. I. Paine

**Affiliations:** 1 Liverpool School of Tropical Medicine, Liverpool, L3 5QA UK; 2 Department of Chemistry, University of Crete, Voutes Campus, Heraklion, 700 13 Greece; 3 Institute of Molecular Biology and Biotechnology, Foundation for Research and Technology, Hellas, 100 N. Plastira Street, Heraklion, 700 13 Greece; 4 Laboratory of Pesticide Science, Department of Crop Science, Agricultural University of Athens, 75 Iera Odos Street, Athens, 118 55 Greece

Correction to: *Scientific Reports* 10.1038/s41598-023-41364-2, published online 29 August 2023

The Acknowledgements section in the original version of this Article was incomplete.

“The work was part funded by the IVCC and Medical Research Council (Grant Ref: MR/V001264/1).”

now reads:

“The work was funded by the IVCC (supported by the Bill and Melinda Gates Foundation under Grant Number OPP1148615), and Medical Research Council (Grant Ref: MR/V001264/1).”

The original Article has been corrected.

