# Supplementary material for: Chlorfenapyr metabolism by mosquito P450s associated with pyrethroid resistance identifies potential activation markers
Source: Sci Rep. 2023 Aug 29;13:14124. doi: 10.1038/s41598-023-41364-2 (PMC10465574; doi:10.1038/s41598-023-41364-2)
Supplement: Supplementary file 1 — Supplementary Information. [file 41598_2023_41364_MOESM1_ESM.pdf]

Supplementary Information

Title: Chlorfenapyr metabolism by mosquito P450s associated with pyrethroid resistance identifies potential activation markers.

Cristina Yunta[1], Jocelyn M. F. Ooi[1], Folasade Oladepo[1], Sofia Grafanaki[2], Spiros. A. Pergantis[2], Dimitra Tsakireli[3,4], Hanafy M. Ismail[1*] and Mark J. I. Paine[1*]

[1] Liverpool School of Tropical Medicine, Liverpool, L3 5QA, UK

[2] Department of Chemistry, University of Crete, Voutes Campus, Heraklion, 733 10, Greece

[3] Institute of Molecular Biology & Biotechnology, Foundation for Research & Technology, Hellas, 100 N. Plastira Street, GR-700 13 Heraklion, Greece

[4] Laboratory of Pesticide Science, Department of Crop Science, Agricultural University of Athens, 75 Iera Odos Street, GR-11855 Athens, Greece

* Co-corresponding authors: mark.paine@lstmed.ac.uk and hanafy.ismail@lstmed.ac.uk

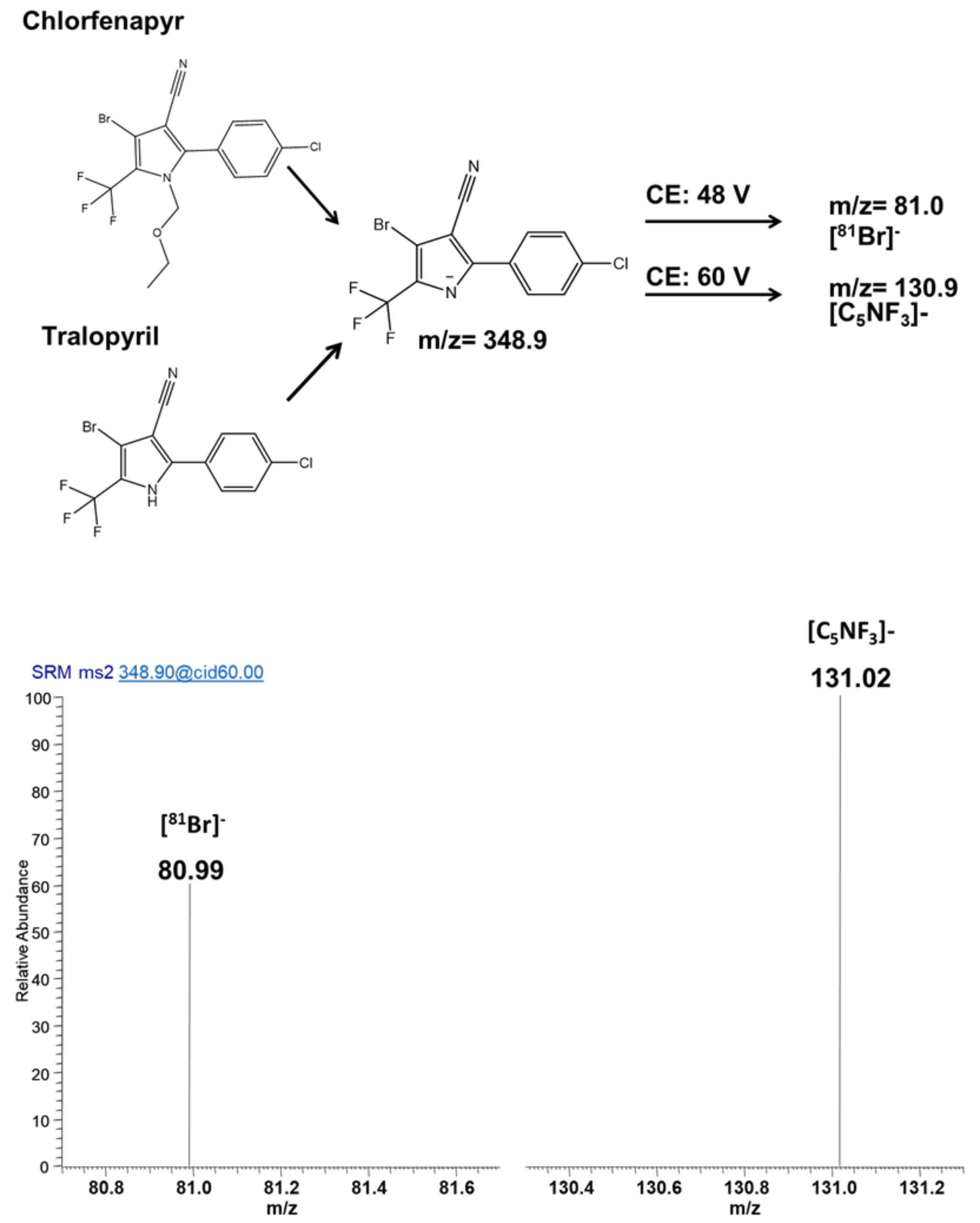

Supplementary Figure 1. Selected reaction monitoring (SRM) transitions for chlorfenapyr and tralopyril after HPLC separation. Selected ion mass spectra obtained from the collision induced dissociation of the precursor ion 348.9 (m/z) for chlorphenapyr and tralopyril. Chlorfenapyr was detected in negative ion mode and not in the protonated molecular ion form, due to the loss of the N-ethoxymethyl group when introduced in the ESI source.

Supplementary Table 1. Chlorfenapyr metabolism

P450	% Chlorfenapyr depletion	
	mean	SE
CYP6M2	nd	nd
CYP6P2	1.2	0.5
CYP6P3	89.8	2.6
CYP6P4	3.5	2.3
CYP6P5	2.4	1.7
CYP9K1	98.4	0.2
CYP9J5	84.2	1.3
CYP9J32	65.4	5.2

nd: not detectable

Supplementary Table 2. Michaelis-Menten kinetics of tralopyril production. K_{cat} is given in pmol product/min/ pmol P450 or min^{-1}, Km in μM and K_{cat}/K_M in $\mu M^{-1} min^{-1}$. Data are mean values ± 95 % confidence intervals (N=4)

P450s	Michaelis-Menten					
	K_{cat}	95% CI	K_m	95% CI	R^2	K_{cat}/K_m
**9J5**	0.59	0.52-0.68	22.81	17.10-31.19	0.97	0.03
**9J32**	1.71	1.55-1.91	16.55	12.73-21.80	0.97	0.10
**9K1**	6.7	6.33-7.11	10.13	8.53-12.06	0.98	0.66
**6P3**	1.71	1.56-1.89	16.32	12.83-20.98	0.97	0.10

Supplementary Table 3. Optimal MS conditions for Chlorfenapyr and Tralopyril

Compound	Parent	Products	Tube Lens (V)	SRM Collision energy (V)	Retention time (min)
Chlorfenapyr	348.9	81	106	48	11.57
		131		60	
Tralopyril	348.9	81	106	48	8.1
		131		60	
